# Phasic Neuronal Firing in the Rodent Nucleus of the Solitary Tract *ex vivo*

**DOI:** 10.3389/fphys.2021.638695

**Published:** 2021-03-02

**Authors:** Lukasz Chrobok, Michal Wojcik, Jasmin Daniela Klich, Kamil Pradel, Marian Henryk Lewandowski, Hugh David Piggins

**Affiliations:** ^1^Department of Neurophysiology and Chronobiology, Institute of Zoology and Biomedical Research, Jagiellonian University, Kraków, Poland; ^2^Faculty of Biology, Medicine and Health, University of Manchester, Manchester, United Kingdom; ^3^School of Physiology, Pharmacology, and Neuroscience, Faculty of Life Sciences, University of Bristol, Bristol, United Kingdom

**Keywords:** brainstem, phasic, multi-electrode array, nucleus of the solitary tract, timekeeping

## Abstract

Phasic pattern of neuronal activity has been previously described in detail for magnocellular vasopressin neurons in the hypothalamic paraventricular and supraoptic nuclei. This characteristic bistable pattern consists of alternating periods of electrical silence and elevated neuronal firing, implicated in neuropeptide release. Here, with the use of multi-electrode array recordings *ex vivo*, we aimed to study the firing pattern of neurons in the nucleus of the solitary tract (NTS) – the brainstem hub for homeostatic, cardio-vascular, and metabolic processes. Our recordings from the mouse and rat hindbrain slices reveal the phasic activity pattern to be displayed by a subset of neurons in the dorsomedial NTS subjacent to the area postrema (AP), with the inter-spike interval distribution closely resembling that reported for phasic magnocellular vasopressin cells. Additionally, we provide interspecies comparison, showing higher phasic frequency and firing rate of phasic NTS cells in mice compared to rats. Further, we describe daily changes in their firing rate and pattern, peaking at the middle of the night. Last, we reveal these phasic cells to be sensitive to *α*_2_ adrenergic receptors activation and to respond to electrical stimulation of the AP. This study provides a comprehensive description of the phasic neuronal activity in the rodent NTS and identifies it as a potential downstream target of the AP noradrenergic system.

## Introduction

Mounting evidence suggests that in addition to the frequency of firing rate, the pattern of action potential generation represents a distinct channel of neuronal information transmission and processing (Buzsáki, 2006; Buzsáki and Llinás, 2017). For example, alternating periods of elevated firing and neuronal silence or “phasic activity” is essential for the secretion of hormones and neuropeptides, providing temporal windows of elevated calcium inflow in axonal boutons and dendrites. This phasic firing has been extensively characterised for the magnocellular vasopressin neurons in the supraoptic and paraventricular nuclei of the hypothalamus (SON and PVN, respectively), where it is necessary for the efficient secretion of vasopressin from the hypophysis (Dutton and Dyball, 1979; Leng et al., 1999; Sabatier et al., 2004; Ludwig et al., 2005; MacGregor and Leng, 2012; Ohbuchi et al., 2015).

The dorsal vagal complex (DVC) of the hindbrain is a major hub for ingestive, cardio-vascular, and homeostatic cues. It consists of (1) the area postrema (AP) – the sensory circumventricular organ situated in the caudal floor of the fourth ventricle, (2) the subjacent nucleus of the solitary tract (NTS), and (3) the dorsal motor nucleus of the vagus (DMV; Grill and Hayes, 2012). Recently, robust circadian timekeeping properties have been documented in the rodent DVC components, with a clear daily and circadian variation in their molecular activities and neuronal firing rate (Herichová et al., 2007; Kaneko et al., 2009; Chrobok et al., 2020; Paul et al., 2020). Although rhythmic neuronal bursting has been observed in the DVC (Tell and Jean, 1991; Paton et al., 2000; Baird et al., 2015), its spontaneous generation and modulation across the daily cycle remain unknown.

Strong connections exist between the AP and NTS in the coronal plane (Morest, 1967; van der Kooy and Koda, 1983; Shapiro and Miselis, 1985; Hay and Bishop, 1991; Abegg et al., 2017), reflecting coronal segmentation observed during the development of the hindbrain (Storm et al., 2008; Bouvier et al., 2017; Kratochwil et al., 2017). Noradrenergic (NA) neurons of the AP extensively innervate the NTS, evoking excitatory and inhibitory responses through selective targeting *α*_2_ adrenergic receptors to effectively modulate synaptic transmission in the NTS (Armstrong et al., 1981; Miceli et al., 1987; Feldman and Felder, 1989; Hay and Bishop, 1991; Aylwin et al., 1998; Potes et al., 2010). As the AP lacks a functional blood-brain barrier, this NA connectivity is considered a conduit for peripheral signals to regulate the NTS (Wang et al., 2008; Potes et al., 2010).

The aim of this study was to describe and characterise the possible patterning of NTS neuronal activity in two rodent species. Here, using the multi-electrode array recordings *ex vivo*, we provide compelling evidence for the phasic neuronal activity to be elicited by a subpopulation of the rat and mouse NTS neurons localised adjacent to the AP. This phasic activity closely resembled the one recorded in the hypothalamic paraventricular nucleus (PVN) and supraoptic nucleus (SON). Additionally, we characterise the phasic NTS cells in rats to undergo daily changes in their firing rate and pattern. Last, by the means of electrophysiological and pharmacological stimulation, we propose these phasic NTS neurons to be a possible target of the AP.

## Materials and Methods

### Animals

All animals were housed under standard (12:12 h) light-dark cycle with the *ad libitum* access to food and water. Environmental conditions were maintained on a constant level (temperature: ~23°C, humidity: ~60%). Experiments were conducted on adult male individuals.

Mice of a C57BL6J genetic background were provided by Charles River, Kent UK and housed in the University of Manchester Biological Services Facility. Sprague Dawley rats were bred in house at the Institute of Zoology and Biomedical Research Animal Facility at the Jagiellonian University in Krakow. Experiments were conducted with approval of (1) Research Ethics committee of the University of Manchester, in keeping with the UK Animal (Scientific Procedures) Act 1986 and (2) Krakow Ethical Commission, in accordance with the European Community Council Directive of 24 November 1986 (86/0609/EEC) and the Polish Animal Welfare Act of 23 May 2012 (82/2012) for mice and rats, respectively.

### Multi-Electrode Array Electrophysiology

#### Tissue Preparation

The majority of experiments was carried out on acute coronal brain slices containing the brainstem NTS: mouse anteroposterior −7.8 to −7.3, mediolateral −1 to +1, dorsoventral −4 to −4.6 (Paxinos and Franklin, 2001); rat anteroposterior −14.3 to −13.7, mediolateral −1.6 to +1.6, dorsoventral −7.2 to −8 mm from Bregma (Paxinos and Watson, 2007). In total, 27 rat brain slices containing NTS were obtained from 21 animals culled at four time points across 24 h (the number of slices in brackets); ZT3 (*n* = 6), ZT9 (*n* = 8), ZT15 (*n* = 6), and ZT21 (*n* = 7). For mice, 24 brain slices containing NTS were obtained from 16 animals culled at two daily time points: ZT0 (*n* = 11) and ZT11 (*n* = 13). Additionally to NTS-containing sections, hypothalamic slices were obtained in order to record from the PVN and the SON, with five mouse brain sections (from three animals, culled at ZT0) and three rat brain sections (from two animals, culled at ZT3). The PVN was collected at anteroposterior −1 to −0.6, mediolateral −0.4 to +0.4, dorsoventral −4.6 to −5 for mice, and anteroposterior −1.9 to −1.5, mediolateral −0.8 to +0.8, dorsoventral −7.6 to −8.6 mm from Bregma for rats. The SON was collected from mice only: anteroposterior −0.9 to −0.7, mediolateral ±1.2 to ±1.4, and dorsoventral −5.6 to −5.4 mm from Bregma.

Animals were deeply anaesthetised in isoflurane and culled by decapitation. Then, brains were quickly removed from the skulls and placed in the ice-cold preparation artificial cerebrospinal fluid (ACSF) composed of (in mM): NaCl 95, KCl 1.8, KH_2_PO_4_ 1.2, CaCl_2_ 0.5, MgSO_4_ 7, NaHCO_3_ 26, glucose 15, sucrose 50, Phenol Red 0.005 mg/L for mice, and NaHCO_3_ 25, KCl 3, Na_2_HPO_4_ 1.2, CaCl_2_ 2, MgCl_2_ 10, glucose 10, sucrose 125, Phenol Red 0.01 mg/L for rat brain slices. ACSF was constantly carbogenated with the mixture of 95% oxygen and 5% CO_2_. Next, brains were appropriately trimmed with a block of tissue containing the structure of interest mounted on a holder and sliced into 250 μm thick coronal sections in an ice-cooled chamber of the vibroslicer (Campden Instruments 7000smz, UK, or Leica VT100s, UK). Whole coronal brainstem slices were harvested with the slice preparation procedure not exceeding 15 min. Sections containing the structure of interest were then transferred to the incubation chamber filled with continuously carbogenated recording ACSF heated to 32°C, composed of (in nM): NaCl 127, KCl 1.8, KH_2_PO_4_ 1.2, CaCl_2_ 2.4, MgSO_4_ 1.3, NaHCO_3_ 26, glucose 5, sucrose 10, Phenol Red 0.005 mg/L for mouse brain slices and NaCl 125, NaHCO_3_ 25, KCl 3, Na_2_HPO_4_ 1.2, CaCl_2_ 2, MgCl_2_ 2, glucose 5, 0.01 mg/L of Phenol Red for rats. Heating was turned off and slices were left in ACSF cooling to the room temperature for an 1–2 h incubation period.

#### Electrophysiological Recordings

After an incubation period, slices were placed in the recording wells of the MEA2100-System (Multichannel Systems GmbH, Germany) with a structure of interest situated above the 6 × 10 perforated multi-electrode recording array (MEA; 60pMEA100/30iR-Ti, Multichannel Systems). Fresh recording ACSF, carbogenated and heated to 32°, perfused the tissue during the entire time of the experiment. Slices were given 30–60 min to settle and then recording was initiated (Belle et al., 2021). First, baseline activity was assessed by recording spontaneous neuronal activity in the slice. Next, drug administrations or electrical stimulation assay were applied. Raw signal was acquired with sampling frequency of 20–25 kHz.

#### Drugs

All drugs: UK 14,304 tartrate (20 μM; Tocris, UK), yohimbine hydrochloride (20 μM; Tocris) were stocked at 100x concentration, stored at −20°C and freshly diluted in the recording ACSF before the application by bath perfusion.

#### Stimulation

For experiments in which the AP was electrically stimulated, we used visual and electrophysiological inspection (higher levels of activity than in the subjacent NTS) to first determine where in the AP the neurons were spontaneously active. Four locations in the central AP were then switched from recording to stimulation mode and electrically stimulated at these selective locations. The previously validated stimulation protocol (Chrobok et al., 2020) was composed of trains of 10 negative voltage pulses (amplitude: 200 mV, duration: 5 ms, inter-stimulus interval: 5 s), repeated three times every 2 min (with 75 s stimulation break). Passive responses to stimulation in recording locations immediately adjacent to the stimulation sites were not recorded.

### Data Analysis and Statistics

#### Data Pre-processing, Spike Sorting, and Phasic Unit Selection

Raw data were exported to HDF5 files with Multi Channel DataManager (Multichannel Systems GmbH) and then processed *via* a custom made MatLab script (R2018a version, MathWorks) to remap and convert the file to DAT format. DAT files were initially automatically spike-sorted with the KiloSort programme (Pachitariu et al., 2016) in the MatLab environment. To enhance the efficiency of spike sorting, a graphics processing unit (NVIDIA GeForce GTX 1050Ti GPU; CUDA 9.0 for Windows) was used. In parallel, raw data were exported to CED-64 files with Multi Channel DataManager, remapped and filtered with Butterworth band pass filter (fourth order) from 0.3 to 7.5 kHz. Spike-sorting data were transferred into the prepared CED-64 files (Spike2 8.11; Cambridge Electronic Design Ltd., United Kingdom) using a custom made MatLab script. The outcome was then checked in Spike2 8.11. First, each putative single unit was inspected by means of autocorrelation and principal component analysis (PCA). Then, if the autocorrelation contained short inter-spike intervals too brief to be attributed to activity of a single neuron (due to refraction period) and/or the PCA displayed significant clustering, these spike sorting results were manually refined. Finally, all channels were explored with the goal of identifying cells with a phasic activity pattern. Phasic units were identified by the inspection of their mean, 1 s binned firing rate – those exhibiting a bimodal frequency distribution and thus firing in discrete periods separated by neuronal silence were classified as phasic.

#### Timestamp-Based Analysis of Neuronal Firing

Inter-spike interval (ISI) histograms, autocorrelograms, hazard plots were calculated in NeuroExplorer 5 (Nex Technologies, United States) with the use of timestamps imported from CED files. All result plots were 10 ms binned. The peri-stimulus histograms of the spike density around 30 stimuli were also generated in NeuroExplorer 5 with the use of Gaussian probability generated with Kernel function (width: 50 ms).

#### Analysis of Firing Patterns and Responses to Drugs

The analysis of firing pattern and responses to pharmacological agents was performed on the 1 s binned data using the custom-written R scripts (R Core Team, 2020). Each recording was divided into two parts: (1) baseline, analysed without modifications and (2) subsequent periods of drug responses. In order to study the maximum potential response of the given unit to the pharmacological treatment, the first 200 s after the drug application were excluded from analysis, as they corresponded with the time necessary for the drug to reach the recording chamber. Therefore, the analysis of the drug response was performed in the subsequent 1,000 s time window. Components of the recordings were further divided into epochs (intrabursts), defined as periods of neuronal activity preceded and followed by bins containing no action potentials (extrabursts). Different parameters describing the baseline and 1,000 s-long responses to a drug administration (Phasic Frequency – number of intrabursts per second, Mean Firing Rate, Maximal Firing Rate), as well as the individual intrabursts (Intraburst Firing Rate, Intraburst length, SD of the Intraburst Firing Rate) were computed. In the latter case, the parameters of all intrabursts were averaged for each part of the recording before further analysis. Neuronal responses to drugs were qualified by means of visual inspection of the 1 s binned recordings.

#### Statistics

All statistical analyses were performed in Prism 7 (GraphPad Software, United States). Interspecies differences and day to night changes in mice were examined with the Mann-Whitney test. Daily changes in rats were analysed with Kruskal-Wallis test. Responses to pharmacological agents were examined with Friedman test followed by Dunn’s multiple comparison. *p* < 0.05 was deemed significant. Data in text were presented as mean ± SEM.

## Results

### Phasic Neuronal Firing in the Nucleus of the Solitary Tract Resembles the Activity Pattern of Putative Vasopressin Magnocellular Hypothalamic Neurons

**Figure 1:**
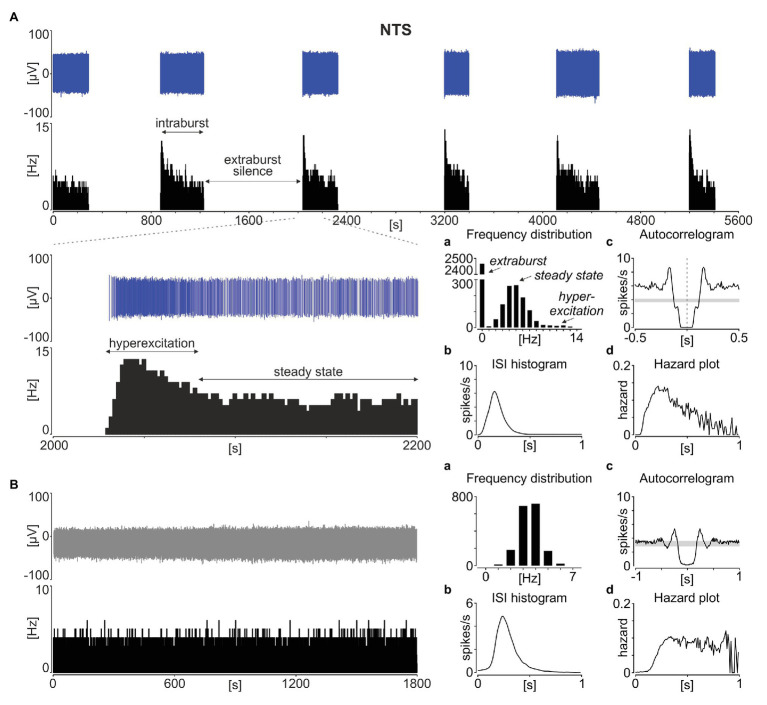
Phasic activity pattern in the rat nucleus of the solitary tract (NTS). **(A)** Spike sorted single units (in blue) with a corresponding firing rate histogram (bin: 1 s) showing a typical phasic activity in the NTS. The enlarged 200 s of the recording is shown below. **(B)** Example of tonic discharge. **(a)** The distribution of firing rates. **(b)** Inter-spike interval (ISI) histograms. **(c)** Autocorrelograms with the 95% CI depicted by the grey box. **(d)** The hazard plot showing the probability of an ISI after each spike. Bins in **(a)** are 1 Hz, while in **(b–d)** – 10 ms.

Neuronal activity in the rodent NTS was recorded *ex vivo* using 60-channel multi-electrode arrays from 51 acute brainstem slices obtained from 37 animals (24 slices from 16 mice and 27 slices from 21 rats). In all of these recordings, a phasic pattern of activity was observed at one or more recording locations; the signal was spike-sorted and further analysed at the single unit activity (SUA) level. Such phasic activity consisted of multi-second long phases of stable neuronal activity separated by a total (or near to complete) electrical silence (Figure 1A). Following a strongly multimodal distribution of frequencies, with the first mode at 0 Hz for the extraburst, the intraburst phase was most often initiated by a short period of hyperexcitation, before progressing to a steady state (Figure 1Aa).

The inter-spike interval histograms calculated for phasic NTS units were broad and unimodal (Figure 1Ab), and the lack of evident regularity in spike timing was also depicted by their autocorrelograms (Figure 1Ac). Therefore, we next calculated the hazard plots, which depict the probability of action potential generation as a function of time from the preceding spike (Figure 1Ad). This enabled us to track how the excitability of these cells changes with the time subsequent to the last spike. Interestingly, the hazard function plotted for phasic NTS neurons was not flat, which would be characteristic for a Poisson (random) distribution of ISIs (Figure 1B), but rather unimodal and right-skewed towards longer ISI values (Figure 1Ad). This deviation from a constant level shows that following the spike generation, there is a period of increased excitability, putatively priming the hyperexcitation at the intraburst phase generation.

**Figure 2:**
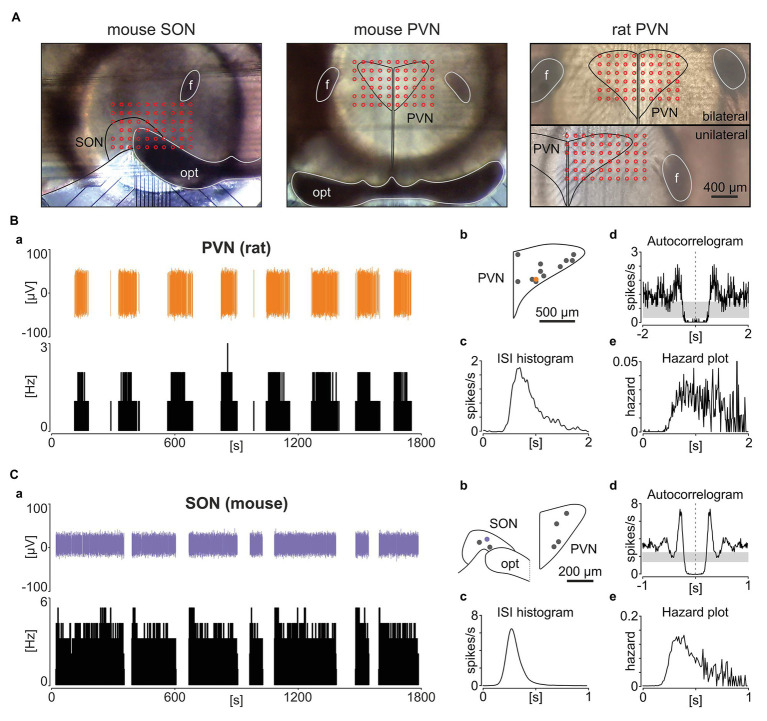
Phasic activity pattern in the rat and mouse hypothalamic paraventricular (PVN) and supraoptic nuclei (SON). **(A)** Photographs showing example hypothalamic slices mounted on the multi-electrode array with the SON and PVN outlined in black and the location of recording electrodes depicted by red circles. opt – optic tract, f – fornix. **(B,C)** The panel **(B)** shows a representative rat PVN phasic unit, whereas **(C)** depicts a phasic cell in the mouse SON. **(a)** Single units with their corresponding firing rate histograms (bin: 1 s). **(b)** Anatomical reconstruction showing the localisation of recorded units. opt – optic tract. **(c)** ISI histograms. **(d)** Autocorrelograms with the 95% CIs coded by grey boxes. **(e)** Hazard plots describing the probability of ISI after each spike. Bins for **(d,e)** are 10 ms.

A similar, if not identical phasic activity pattern was previously described for hypothalamic vasopressin neurons in the PVN and SON (Sabatier et al., 2004; MacGregor and Leng, 2012). To directly determine how closely phasic NTS activity resembles these hypothalamic neurons, we recorded and analysed five coronal hypothalamic slices from three mice and three slices from two rats with the same experimental setup as used in our brainstem recordings (Figure 2A). We found seven and 12 neurons following the phasic activity pattern in mouse and rat recordings, respectively, in the area of the PVN or SON. This phasic activity resembles the bimodal frequency distribution described by Sabatier et al. (2004). Additionally, these putative vasopressin neurons fired action potentials with the same ISI distribution to phasic NTS cells, mirroring their autocorrelation and hazard function (Figures 2B,C), in keeping with aforementioned reports. Therefore, phasic neuronal activity in the rodent brainstem shares many similarities to the activity pattern exhibited by the hypothalamic magnocellular vasopressin neurons.

### Phasic Neuronal Activity Differs Between Mice and Rats

**Figure 3:**
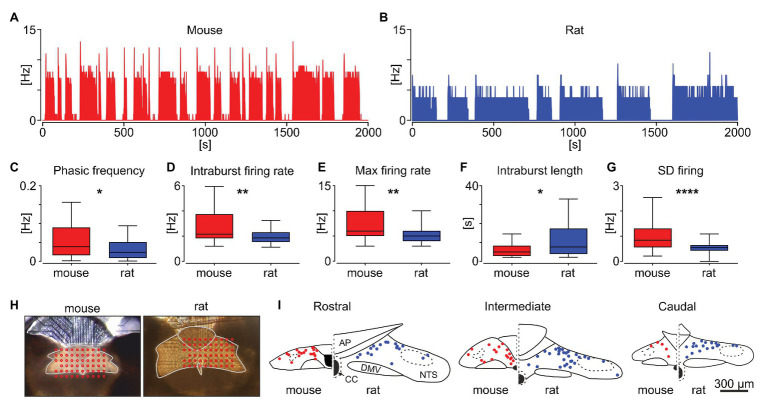
Comparison of phasic activity pattern in the mouse and rat NTS. Example phasic firing rate histograms (bin: 1 s) for mouse **(A)** and rat **(B)**. **(C–G)** Statistical comparison of the frequency of phasic burst occurrence (phasic frequency), intraburst firing rate, maximum firing rate, intraburst length, and the variability of firing within the intraburst (shown as the SD of firing). The horizon line within the box is median value, while the box indicates interquartile range. Whiskers include all data points. ^*^p < 0.05, ^**^p < 0.01, ^****^p < 0.0001, Mann-Whitney tests. **(H)** Photographs of brainstem slices mounted on the multi-electrode array with the dorsal vagal complex outlined in grey and the location of recording electrodes shown in red. **(I)** Anatomical reconstruction of the recorded phasic cells in the mouse (in red) and rat NTS (in blue). AP – area postrema, CC – central canal, DMV – dorsal motor nucleus of the vagus.

Next, we investigated possible species differences in the characteristics of the phasic activity pattern in the NTS (Figures 3A,B) by comparing 40 phasic units in mouse and 65 in the rat brainstem. The number of intraburst phases per unit of time (named the phasic frequency) was significantly higher for units recorded in mice, compared to rats (0.052 ± 0.006 Hz vs. 0.031 ± 0.003 Hz, *p* = 0.0115; Figure 3C). Also, the intraburst firing rate and maximal firing rate were notably higher in mice (*p* = 0.0053 and *p* = 0.0048, respectively; Figures 3D,E). The higher phasic frequency in mice was explained by the significantly shorter intraburst phases (*p* = 0.0123; Figure 3F). Accompanying the higher firing rate, the variability of neuronal firing within the intraburst, measured as a SD of the 1 s binned firing rate, was clearly elevated for mouse units, compared to rat (*p* < 0.0001, Mann-Whitney tests; Figure 3G). Subsequently, the location of all units recorded was extracted and mapped on the NTS outline (Figure 3H). These reconstructions showed that in both species, the neurons exhibiting phasic firing were predominantly localised in dorsomedial NTS, adjacent and subjacent to the AP (Figure 3I). These results provide evidence that the phasic neuronal activity in the NTS varies between rodent species, with mouse units characterised by higher rate, phasic frequency and more variable firing to those recorded in rats.

### Phasic Activity Is Not Periodic nor Synchronised Amongst NTS Neurons

**Figure 4:**
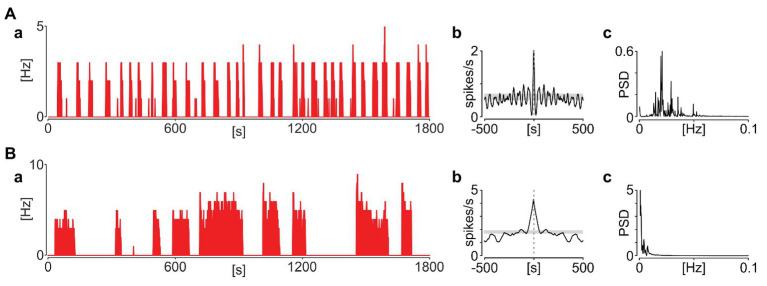
Phasic activity pattern in the NTS is rarely periodic. **(A)** Example of a rare periodic unit. **(B)** Representative non-periodic activity. **(a)** Firing rate histograms (bin: 1 s). **(b)** Autocorrelograms (bin: 1 s). Grey boxes code 95% CIs. **(c)** Periodograms. PSD – power spectral density.

**Figure 5:**
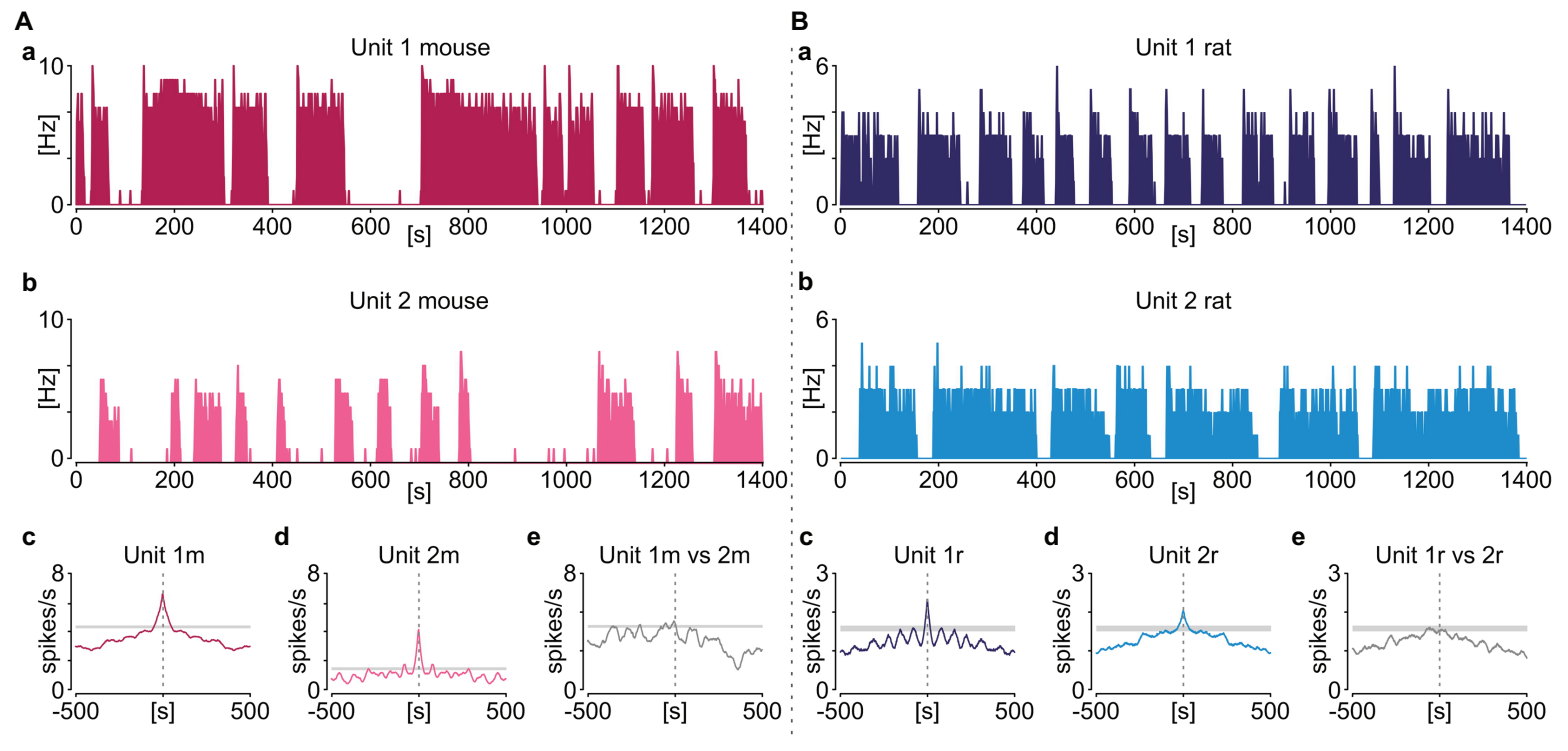
Phasic activity in the NTS is not synchronised amongst its neurons. Example pairs of simultaneously recorded NTS neurons displaying phasic activity pattern in mouse **(A)** and rat **(B)**. **(a,b)** Firing rate histograms for individual neurons (bin: 1 s). **(c,d)** Corresponding autocorrelograms (bin: 1 s). **(e)** Cross correlation between two units. 95% CIs are coded by grey boxes.

This repetitive sequence of elevated SUA followed by the extraburst silence was sustained throughout the recording, but without being overtly periodic (as would be expected from an oscillatory process). In detail, only four out of 65 phasic neurons in rats and seven out of 33 in mice were classified as rhythmic based on significant periodicity in their autocorrelograms and a distinct peak in periodograms (Figure 4). Additionally, when more than one phasic neuron was recorded simultaneously in the same slice, their activity was not synchronised (Figure 5).

### Daily Changes in Phasic Activity Pattern

**Figure 6:**
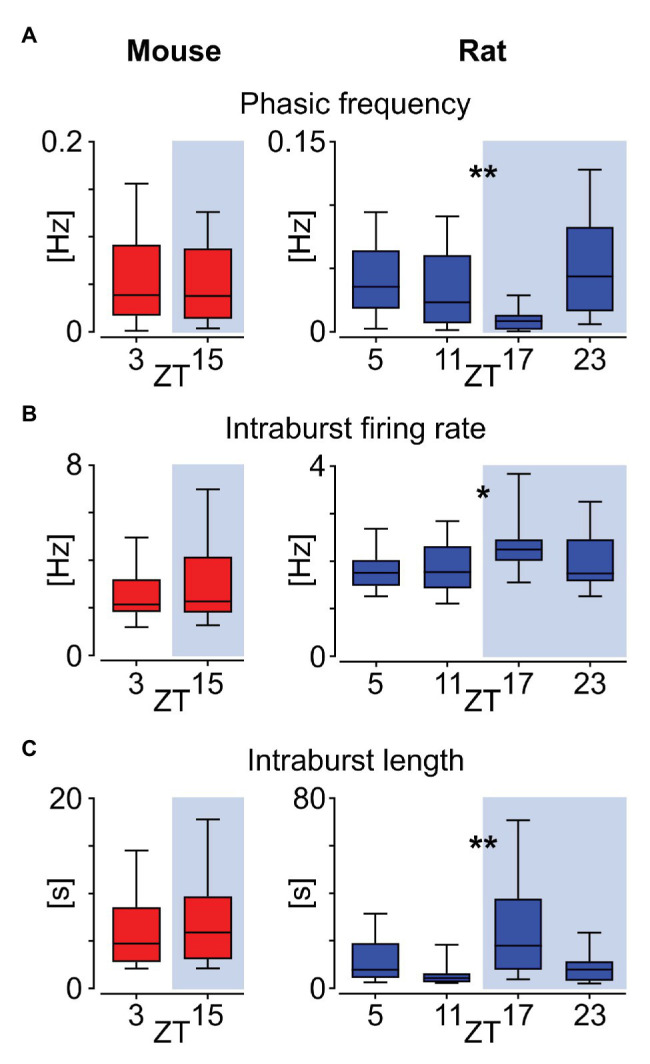
Daily variation in the phasic activity pattern in the rat but not mouse NTS. **(A)** Frequency of phasic bursts occurrence. **(B)** Firing rate within the intrabursts. **(C)** Mean intraburst length. All comparisons were calculated with Mann-Whitney tests for mice and Kruskal-Wallis tests for rat data. The horizon line within the box is median value, while the box indicates interquartile range. Whiskers include all data points. ^*^p < 0.05, ^**^p < 0.01. ZT – Zeitgeber time.

Neuronal activity in the murine DVC exhibits daily and circadian changes, with higher levels at late day/early night (Chrobok et al., 2020). Thus, here we assessed if phasic neurons in the NTS are amongst these potential timekeeping neurons. The comparison of recordings performed on mouse slices near the beginning of light and dark phases (ZT3 and 15, respectively) showed no day to night variation in the frequency of phasic bursts appearance (*p* = 0.6535; Figure 6A), nor in the intraburst firing rate (*p* = 0.4430; Figure 6B) and intraburst length (*p* = 0.5508, Mann-Whitney tests; Figure 6C). However, the higher temporal resolution of rat recordings revealed daily alteration in these measured parameters. Namely, the rat phasic activity pattern exhibited daily variation in phasic frequency (*p* = 0.0025; Figure 6A), intraburst firing rate (*p* = 0.0235; Figure 6B) and intraburst length (*p* = 0.0048, Kruskal-Wallis tests; Figure 6C). Both the mean firing rate within intrabursts and their length peaked in the middle of the night (ZT17), at the time of the lowest frequency of phasic events. These observations indicate that phasic cells in the rat NTS increase their firing rate and elongate intraburst phase at night, which is accompanied by the less frequent switching between activity and silent phases.

### Phasic Neurons in the Nucleus of the Solitary Tract Are Sensitive to *α*_2_ Adrenergic Compounds and Electrical Stimulation of the AP

**Figure 7:**
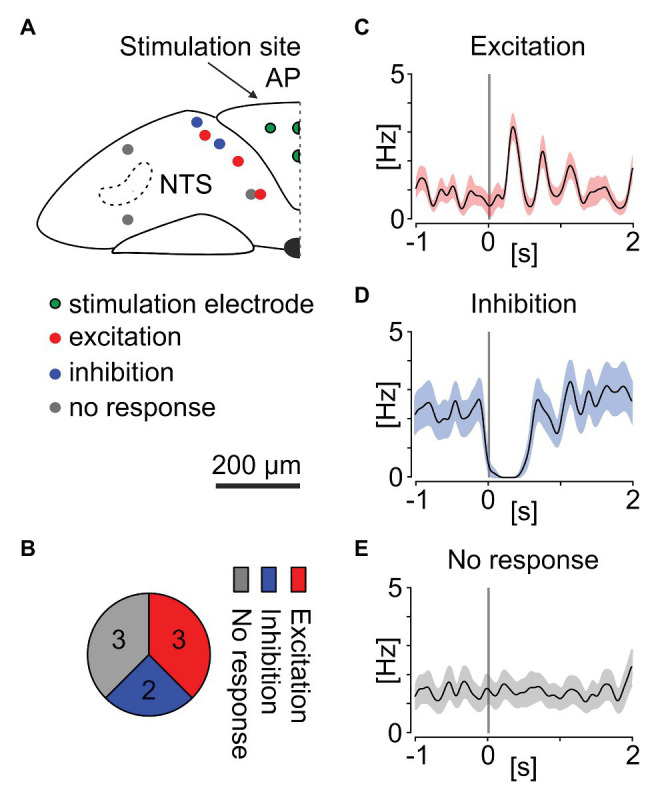
Electrical stimulation of the AP alters neuronal activity of phasic neurons in the mouse NTS. **(A)** Anatomical reconstruction showing the localisation of the stimulation electrodes in the AP (green circles) and phasic cells in the NTS: excited by the AP stimulation (red), inhibited (blue), or exhibiting no response (grey circles). **(B)** Pie chart summarising the proportion of responsive cells. **(C–E)** Mean peri-stimulus firing rate plots showing the spike density of all excited, inhibited, and non-responsive cells before and after the AP stimulation. Gaussian probability was generated with Kernel function (width: 50 ms). Grey bar depicts the stimulation time. Shading codes the average SEM for all responses of all neurons in one group.

Noradrenergic neurons of the AP extensively innervate the NTS in the coronal plane, reflecting the conserved developmental plan in the brainstem (Abegg et al., 2017; Kratochwil et al., 2017). The AP to NTS neuronal connection is preserved *ex vivo* and can be examined through electrical stimulation protocols (Hay and Bishop, 1991). Thus, we first evaluated if the phasic NTS neurons receive electrical input from the AP. In four separate experiments performed on four mouse brainstem slices, four recording electrodes of the MEA localised in the centre of the AP were switched to stimulation mode. Then, three trains of 10 negative voltage pulses (amplitude: 200 mV, duration: 5 ms, inter-stimulus interval: 5 s), repeating every 2 min (a 75 s stimulation break) were delivered *via* the stimulation site and neuronal responses in the NTS were recorded and further analysed. We found eight NTS neurons exhibiting the phasic activity pattern, most of which localised immediately subjacent to the AP (Figure 7A). The mean peristimulus firing rate plots based on the spike density function around 30 stimuli indicated that out of eight phasic NTS units, three were notably excited (Figures 7B,C) and two profoundly inhibited by the AP stimulation (Figures 7B,D). The activity of two remaining phasic cells remained unchanged following the electrical stimulation (Figures 7B,E).

**Figure 8:**
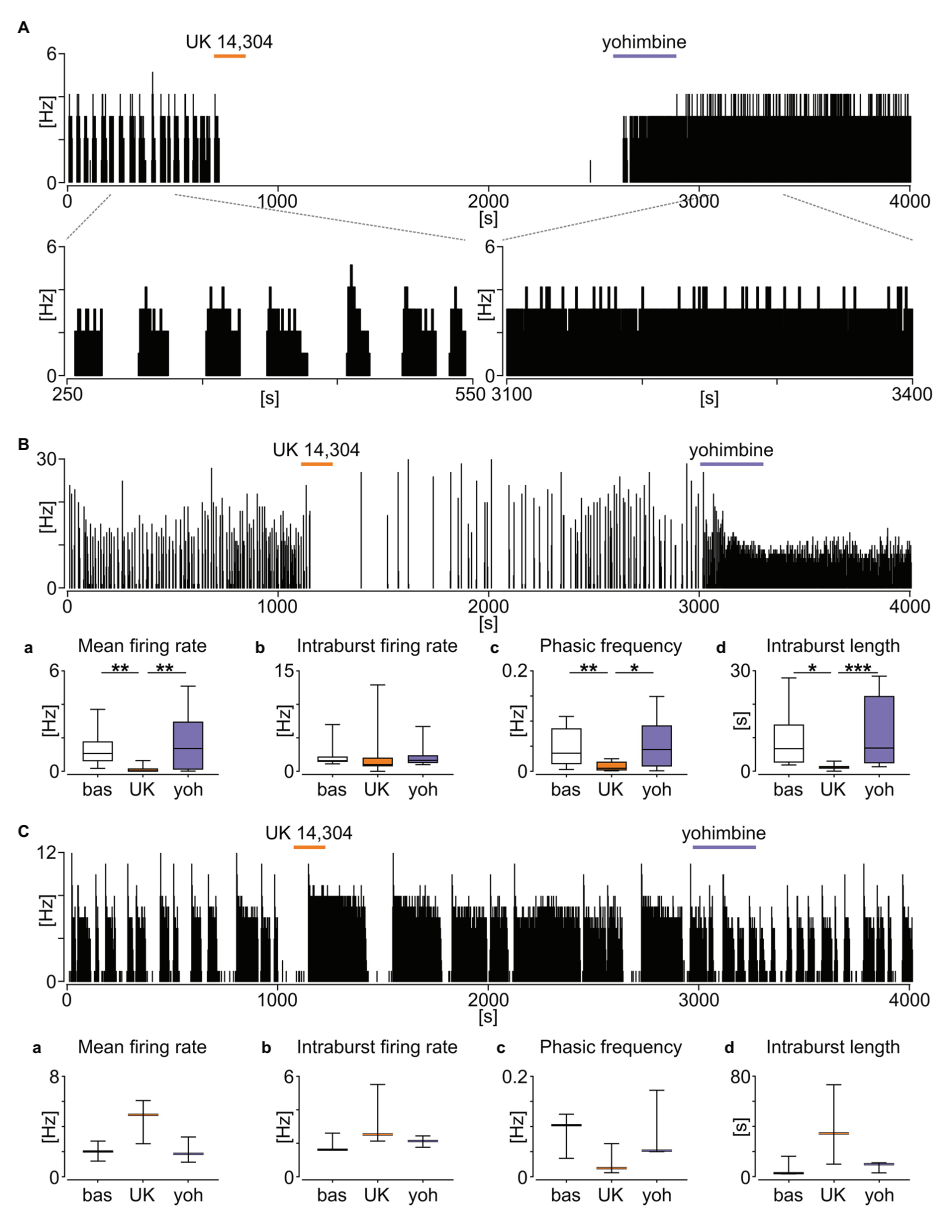
Phasic neurons in the NTS are sensitive to the activation of *α*_2_ adrenergic receptors. **(A)** Firing rate histogram (bin: 1 s) showing the example effect of *α*_2_ adrenergic receptor stimulation with an agonist UK 14,304 (in orange) causing a total silencing of neuronal activity, restored by an antagonist/inverse agonist yohimbine (in purple). The subpanel below shows an enlarged 300 s fragment of the baseline and response to yohimbine. **(B)** Representative recording of the phasic activity decreased by the UK 14,304 and rebounded after yohimbine. **(Ba–d)** Statistical summary of the changes in the phasic activity pattern induced by *α*_2_ adrenergic receptor compounds. The horizon line within the box is median value, while the box indicates interquartile range. Whiskers include all data points. ^*^p < 0.05, ^**^p < 0.01, ^***^p < 0.001, Dunn’s multiple comparison tests. **(C)** Example of the phasic unit excited by the UK 14,304, which activity was restored after yohimbine application. **(Ca–d)** Summary of the drug-evoked changes. No statistical analysis was performed due to a small group size (*n* = 3). The horizon line depicts median values. Whiskers include all data points.

Noradrenergic signalling from the AP to NTS utilises *α*_2_ adrenergic receptors, creating a distinct connection between these structures (Aylwin et al., 1998). Therefore, we next investigated if pharmacological agents activating or inhibiting *α*_2_ adrenergic receptors modulate the neuronal activity of phasic NTS neurons. Here, we used eight brainstem slices from five mice, in which we found 15 neurons exhibiting a phasic firing pattern in their SUA. First, we applied the *α*_2_ receptor agonist UK 14,304 (20 μM), which notably affected the neuronal activity of ~87% (13/15) of tested units. Marked inhibition of the neuronal activity (noted in 10/13 responsive neurons) was seen including transient total silencing of SUA (6/13; Figure 8A) or its profound decrease (*p* = 0.0052; Figure 8Ba). This suppression of firing was not accompanied by significant changes in the intraburst firing rate (*p* = 0.1009; Figure 8Bb), but rather by the lowering of phasic burst occurrence frequency (*p* = 0.0016; Figure 8Bc) and shortening of the intraburst phase (*p* = 0.0286, Dunn’s multiple comparison tests; Figure 8Bd). A minority of responsive units (3/10) increased their mean firing rate by broadening their intraburst phase and thereby lowering phasic frequency (Figure 8C). Subsequently, we applied yohimbine (20 μM; an *α*_2_ receptor antagonist with an inverse agonist action) to the same neurons. Yohimbine elicited the opposite response to that of UK 14,304 application, with neuronal activity either restored to near baseline firing or altered to a more tonic discharge pattern (Figures 8A,B). When compared to UK 14,304-evoked silencing, with treatment with yohimbine, the mean firing rate (*p* = 0.0010; Figure 8Ba), phasic frequency (*p* = 0.0110; Figure 8Bc), and intraburst length (*p* = 0.0005; Figure 8Bd) significantly rebounded to near baseline-like parameters (*p* > 0.9999, *p* > 0.9999, and *p* = 0.7158, respectively, Dunn’s multiple comparison tests; Figures 8Ba–d). Yohimbine was also potent in rescuing the activity of units activated by UK 14,304 (Figure 8C). These findings indicate that NTS neurons displaying phasic activity pattern are a potential downstream target of AP neurons and are responsive to the activation of *α*_2_ adrenergic receptors.

## Discussion

Here, we provide compelling evidence that phasic neuronal firing by a subpopulation of mouse and rat NTS neurons closely resembles the firing pattern observed for the vasopressin cells of the hypothalamic SON and PVN. Interestingly, the intraburst firing rate, intraburst length, and phasic frequency of this activity differ between these two rodent species. Additionally, with the use of electrophysiological and pharmacological approaches, we reveal these phasic cells to be a potential target of the NA AP neurons.

Phasic firing patterns have been extensively studied both *in vivo* and *ex vivo* for the magnocellular vasopressin cells (for review see: MacGregor and Leng, 2016). This distinct phasic activity comprises alternating periods of neuronal excitation and electrical silence, with a characteristic bimodal frequency distribution. Despite a short hyperexcitation at the beginning of the phasic discharge, these neurons show preference for a certain intraburst firing rate, which they maintain throughout the steady state. Therefore, the distinguishing rectangular (bistable) shape of the firing rate histogram for phasic neurons differs substantially from other oscillatory and bursting cells relying on different ionic mechanisms of pattern generation (Buzsáki, 2006). Here, we find this distinct phasic discharge in a subpopulation of brainstem NTS neurons, whose firing properties closely resemble those described in the hypothalamus. Our investigation was performed in acute brainstem slices *ex vivo*, thus it is likely that observed phasic patterning of neuronal activity stems from mechanisms intrinsic to the DVC. However, we cannot rule out the possibility that in the absence of efferent information from extra-brainstem sites this pattern could differ from that recorded *in vivo* from freely moving rodents.

The proposed mechanism for phasic activity of the magnocellular neurons comprises the relay of subsequent ionic conductances. First, after a single spike generation, there is an immediate period of inexcitability named the *hyperpolarising afterpotential* (HAP), mediated by the activation of voltage and Ca^2+^ dependent K^+^ channels. However, what is crucial for the initiation of phasic burst, this is followed by a slower *depolarising afterpotential* (DAP), leading to the hyperexcitability, which triggers the subsequent firing. The intraburst terminates when the accumulation of Ca^2+^ activates the long lasting reduction of excitability identified as the *afterhypolarisation* (AHP; Andrew and Dudek, 1983; Armstrong et al., 1994; Roper et al., 2003, 2004; Sabatier et al., 2004). Importantly, any alteration in neuronal excitability can be plotted as the hazard function, which shows the probability of the spike generation as a function of time elapsed since the last spike. We show that the hazard plots for these NTS neurons which exhibit phasic activity pattern do not differ from magnocellular vasopressin cells recorded in our study and by others (Sabatier et al., 2004); the enhanced probability of relatively short ISIs represents the DAP-evoked hyperexcitation. Interestingly, the constant probability depicted by the plateau of the hazard function calculated for non-phasic NTS neurons resembles that reported for tonic oxytocin cells (Sabatier et al., 2004). Therefore, the current findings raise the possibility that these anatomically separated brainstem and hypothalamic neurons share similar mechanisms of discharge pattern generation.

Our study explores the phasic activity pattern in the NTS of two rodent species – mouse and rat. The striking similarities in the pattern characteristics and the anatomical localisation of the phasic cells in these two species enabled us to classify these units to be the same neuronal subpopulation. However, phasic NTS cells displayed some interspecies differences. In mice, these neurons were characterised by a higher intraburst firing rate, and more frequently occurring but shorter intraburst phases, compared to rats. Considering the same mechanism of phasic activity generation between the NTS and hypothalamus, this interspecies difference cannot be attributed to the membrane potential alone, as the previous reports from the magnocellular vasopressin neurons show that the depolarisation or increase in the synaptic input causes the reduction of phasic frequency accompanied by the prolongation (and not shortening) of the intraburst, without any change in the intraburst rate (Andrew and Dudek, 1984; Bourque and Renaud, 1990). Notably, the frequency of many rhythmic processes in mammals is inversely proportional to the body size; the smaller the species the higher the metabolic rate. This principle applies to differences amongst rodents but also to the comparison of rodent species with human (Ringwood and Malpas, 2001; Jochmans-Lemoine et al., 2015; Janssen et al., 2016; Jacobs et al., 2017). Therefore, our findings provide a further insight to comparative neurophysiology.

Intriguingly, we found that phasic activity pattern was not evenly distributed in the NTS – most of phasic neurons were localised in its dorsomedial part, in proximity to the AP. Our previous study found that in mice, this particular subdivision of the NTS has direct access to blood-borne information during the behaviourally active night, when the glial barrier between the AP and the NTS exhibits notable permeability (Chrobok et al., 2020). Other studies localise aldosterone-sensing cells or neuropeptide Y-synthesising neurons in this NTS subdivision (van den Pol et al., 2009; Resch et al., 2017). However, due to the heterogeneous nature of NTS cells, we are unable to definitely indicate the biochemical type of the phasic neurons based on their localisation in the structure. Further research using genetically modified animals bearing reporter constructs is necessary to help address this.

Functionally, phasic firing has been implicated in the release of neuropeptides; vasopressin released from terminals of SON neurons in the pituitary clearly relies on the clustered rather than tonic train of action potential reaching the axonal boutons (Harris et al., 1975; Dutton and Dyball, 1979; Bisset and Chowdrey, 1988; MacGregor and Leng, 2012). Interestingly, previous investigations demonstrated that phasic activity is highly efficient in peptide release, but this effectiveness does not stem from the abundance of short ISIs in the intraburst phase alone. Rather, the elevated activity leading to Ca^2+^ influx, fluctuating with the recovery silence is the most effective firing pattern for peptide secretion (Bicknell and Leng, 1981). Since the temporal parameters in the phasic firing of NTS neurons described here are similar to those of the neuropeptide secreting magnocellular PVN and SON cells, we speculate that such patterns of discharge could serve to enable release of peptides such as cholecystokinin and neuropeptide Y from this subpopulation of NTS neurons (van den Pol et al., 2009; D’Agostino et al., 2016). Further studies are required to interrogate this possibility.

Accumulating evidence place the DVC, including the AP and NTS, as an important hindbrain circadian timekeeping centre (Kaneko et al., 2009; Ubaldo-Reyes et al., 2017; Chrobok et al., 2020). Interestingly, in our previous study, we identified an area of the NTS adjacent to the AP in which circadian rhythmicity in the *Per2* clock gene expression was sustained in *ex vivo* brain slices for up to a week in culture. From this, we concluded that the NTS is a circadian oscillator that can function independently of the master circadian clock in the suprachiasmatic nuclei. Additionally, we determined that AP and NTS neurons display daily changes in the firing rate, with peak firing occurring late day/early night (Chrobok et al., 2020). In the current study, we report daily changes in the firing pattern characteristics of phasic neurons in the rat NTS with phasic frequency decreasing around the middle of the night (ZT17), along with an increase in their intraburst firing rate and length. This daily variation may reflect the intrinsic ability of these phasic NTS neurons to regulate their firing from day to night, or stem from the input from another circadian oscillator such as the AP. Based on the studies on phasic magnocellular neurons (Andrew and Dudek, 1984; Bourque and Renaud, 1990), we hypothesise that this change in firing pattern reflects neuronal activation arising from membrane depolarisation. In our investigation, we did not detect significant day-to-night differences in the firing pattern of mouse phasic NTS cells and this may represent a bona fide species difference. However, a caveat here is that with sampling at only two phases, we cannot rule out the possibility that mouse NTS neurons can vary in phasic firing mode at other phases of the 24 h cycle. An additional limitation is that the cull and/or the slice preparation procedure could affect the phase of the daily neuronal activity (Guilding et al., 2009) in the NTS in a species specific manner.

The physiological role of phasic NTS cells is yet to be determined, but the results of our electrophysiological and pharmacological investigations raise the possibility that these neurons receive AP-derived signals. A plethora of evidence describes the highly organised AP-NTS neuronal pathway; the NA neurons of the AP that predominately co-release glutamate, innervate the NTS on the coronal plane to modulate the activity of the targeted neurons *via* activation of *α*_2_ adrenergic receptors (Morest, 1967; Armstrong et al., 1981; van der Kooy and Koda, 1983; Shapiro and Miselis, 1985; Miceli et al., 1987; Feldman and Felder, 1989; Hay and Bishop, 1991; Roth et al., 2010; Abegg et al., 2017). Here we show that the majority of the phasic mouse NTS neurons altered their firing in response to the electrical stimulation of the AP. However, the variability in the response latency and the clear difference between the timing of excitatory and inhibitory response does not allow us to assess if the response to AP stimulation was direct, or rather a result of indirect, network mechanisms. It should be also noted that the lack of response *ex vivo* does not necessarily indicate the lack of connectivity *in vivo*, as it is possible that some of this connectivity is absent in the slice preparation. It is clear from our study that nearly all phasic NTS cells are sensitive to *α*_2_ adrenergic receptor activation. These neuronal responses were mostly inhibitory, but we also recorded rare neuronal activations, manifested in the lengthening of the intraburst phase together with the decrease in the frequency of phasic events (Andrew and Dudek, 1984). Collectively, this implies that phasic NTS neurons receive and process the AP-derived information, which may include the response to osmotic, metabolic, or chemical challenges (Adachi et al., 1991; Miller and Leslie, 1994; McKinley et al., 2003; Johnstone et al., 2006; Abegg et al., 2017).

In conclusion, this study describes a phasic activity pattern of rodent NTS neurons, which can vary with time of day and is influenced by input from the AP. Additionally, due to a similarity of these cells to the firing patterns of magnocellular vasopressin neurons, we speculate that the neurophysiological role of phasic activity in the brainstem is for promoting neuropeptide release. This study also describes interspecies differences in a fundamental neurophysiological phenomena.

## Data Availability Statement

The raw data supporting the conclusions of this article will be made available by the authors, without undue reservation.

## Ethics Statement

The animal study was reviewed and approved by 2nd Local Ethical Commission in Krakow, Poland; Research Ethics Committee of the University of Manchester, United Kingdom.

## Author Contributions

LC and HP conceived the project. LC and JK performed the recordings. JK spike-sorted the data. MW created custom-made scripts in R and analysed the data. KP optimised spike-sorting and provided custom-made tools. LC with the help of JK and MW wrote the first version of the manuscript. LC, ML, and HP provided financial support. All authors contributed to the article and approved the submitted version.

### Conflict of Interest

The authors declare that the research was conducted in the absence of any commercial or financial relationships that could be construed as a potential conflict of interest.
